# Supplementary material for: Endocrine therapy reprogramming of breast cancer facilitates metastatic escape via upregulation of P-Rex1/Rac1 signalling
Source: Nat Commun. 2026 May 11;17:3042. doi: 10.1038/s41467-026-70683-x (PMC13161276; doi:10.1038/s41467-026-70683-x)
Supplement: Supplementary file 1 — Supplementary Information [file 41467_2026_70683_MOESM1_ESM.pdf]

SUPPLEMENTARY MATERIALS FOR:

Endocrine therapy reprogramming of breast cancer facilitates metastatic escape via upregulation of P-Rex1/Rac1 signalling

Kristine J. Fernandez*[1], Ghazal Sultani*[1,2], Max Nobis[3], Brian Gloss[4], Leila Eshraghi[1,2], Amy E. McCart Reed[5], Sarah Alexandrou[1,2], Christine Lee[1], Daniel L. Roden[1,2], Emily I. Jones[6], Maryam Hasha Simad[1,2], Ewan K. A. Millar[7,8], Nenad Bartonicek[1], Samantha R. Oakes[2,9,10], Fatima Valdes-Mora[2,11], Yolanda Colino-Sanguino[11], Ellie T.Y. Mok[1,2], Hannah L. Williams[1,12], Jamie R. Kutasovic[5], Margaret C. Cummings[5,13], Janett Stoehr[1], Victoria Lee[1], Kate Harvey[1], Sunny Wu[1,2], Sunil R. Lakhani[5], Peter T. Simpson[5], Thomas R. Cox[1,2], Lisa M. Ooms[6], Christina A. Mitchell[6], Rob Salomon[14], Alexander Swarbrick[1,2], David Gallego-Ortega[1,2,15], Elgene Lim[1,2,16], Paul Timpson[1,2], C. Elizabeth Caldon[1,2^]

* equal contribution
^ corresponding author

1. Garvan Institute of Medical Research, Sydney, NSW 2010, Australia
2. School of Clinical Medicine, Faculty of Medicine and Health, UNSW Sydney, NSW 2052, Australia
3. Manchester Cell-Matrix Centre, Division of Cell Matrix Biology and Regenerative Medicine, School of Biological Sciences, Faculty of Biology Medicine and Health, The University of Manchester, Manchester, United Kingdom
4. Westmead Research Hub, Westmead Institute for Medical Research, Westmead, NSW 2145, Australia
5. The University of Queensland, Faculty of Health, Medicine and Behavioural Sciences, Centre for Clinical Research, Brisbane, QLD 4029, Australia
6. Cancer Program, Monash Biomedicine Discovery Institute and Department of Biochemistry and Molecular Biology, Monash University, Victoria 3800, Australia
7. St George and Sutherland Clinical Campuses, School of Clinical Medicine, UNSW Medicine and Health, UNSW Sydney, Sydney, NSW 2052, Australia
8. Department of Anatomical Pathology, NSW Health Pathology, St George Hospital, Kogarah, NSW 2217, Australia
9. ANZUP Cancer Trials Group, Health Translation Hub, Level 8, 55 Botany Street, Randwick, Sydney, NSW 2031, Australia
10. The George Institute for Global Health, Health Translation Hub, Level 8, 55 Botany Street, Randwick, Sydney, NSW 2031, Australia
11. Cancer Epigenetic Biology and Therapeutics, Therapeutic Discovery Theme, Children's Cancer Institute, Sydney, NSW 2031, Australia
12. Institute for Tissue Medicine and Pathology, University of Bern, Bern, Switzerland
13. Pathology Queensland, The Royal Brisbane and Women's Hospital, Brisbane, QLD 4006, Australia
14. Institute for Biomedical Materials and Devices, University of Technology Sydney, Sydney, NSW 2007, Australia
15. School of Biomedical Engineering, Faculty of Engineering and IT, University of Technology Sydney, Ultimo, NSW 2007, Australia
16. St Vincent's Hospital, Darlinghurst, Sydney, NSW 2010, Australia

Supplementary Table 1 - Characteristics of PDX models screened in Supplementary Figure 8A

Model	Reference	Hormone receptor status	Prior treatment	P-Rex1 H-score
HCI-004	Rose et al (2011)	ER-/PR-	None	0.24
HCI-006	Rose et al (2011)	ER+/PR+	doxorubicin; cyclophosphamide; tamoxifen; letrazole; zolendronic acid; fulvestrant; capecitabine; trastuzumab; vinorebline; paclitaxel; liposomal doxorubicin; gemcitabine; carboplatin	70.51
HCI-009	Rose et al (2011)	ER-/PR-	cyclophosphamide; methotrexate; 5-fluorouracil; tamoxifen; anastrozole; paclitaxel; zolendronic acid; fulvestrant	16.96
HCI-011	Rose et al (2011)	ER+/PR+	doxorubicin; cyclophosphamide; paclitaxel; fulvestrant	25.17
GAR1513D	Chia et al (2019)	ER+/PR-	chemotherapy + adjuvant radiotherapy; Arimidex	7.41
3837 FasR	Alves et al (2021)	ER+/PR+	Patient treatment naïve; PDX chronically treated with fulvestrant	4.70
3837 FasRPalbR	Alves et al (2021)	ER+/PR+	Patient treatment naïve; PDX chronically treated with fulvestrant and palbociclib	5.76
KCC4653	this manuscript	ER+/PR+	Adriamycin and cyclophosphamide, tamoxifen, letrozole, denosumab, exemestane, capecitabine, methotrexate, abemaciclib	134.63

Supplementary Table 2 - Studies in Meta-Analysis

	Study	Year	Drug	Dose	Analgesic	Control/other treatment	Hazard ratio used in analysis
Ketorolac	**Desmedt**	2018	Ketorolac	20 mg in patients <60 kg and 30 mg in patients ≥60 kg	529	298	HR: 0.59; 95% CI: 0.37-0.96; p = 0.03
	**Forget**	2019	Ketorolac	30 mg	96	107	HR: 1.23; 95% CI: 0.65-2.31; P<0.517
	**Forget**	2014	Diclofenac (29%) and ketorolac (71%)	ketorolac : 20 mg in patients <60 kg and 30 mg in patients ≥60 kg; or diclofenac: 75 mg	510	210	HR: 0.56; 95% CI: 0.36-0.86; P<0.009
	**Forget**	2010	Ketorolac	20 mg	175	144	HR: 0.37; 95% CI:0-0.79; P<0.0.019
	**Forget**	2013	Ketorolac	20 mg in patients <60 kg and 30 mg in patients ≥60 kg	82	80	HR: 0.38; 95% CI: 0.07-1.95; P = 0.24
	**Forget**	2013	Diclofenac (19%) and ketorolac (81%)	ketorolac : 20 mg in patients <60 kg and 30 mg in patients ≥60 kg; or diclofenac: 75 mg	112	60	HR: 0.17; 95% CI: 0.07–0.43, P = 0.0002
	**Study**	**Year**	**Drug**	**Dose**	**Analgesic**	**Control/other treatment**	**Hazard ratio**
Non-GTPase analgesic	**Desmedt**	2018	Diclofenac	75 mg	788	221	HR: 1.04, 95% CI: 0.58-1.87, P = 0.88
	**Forget**	2010	Sufentanil	15 µg	227	92	HR: 0.73; 95% CI: 0-1.83 P<0.57
	**Forget***	2010	Ketamine	10 mg	166	153	HR:0.57; 95% CI: 0-1.49 P<0.23
	**Forget***	2010	Clonidine	150 µg	184	135	HR:2.17; 95% CI: 0.33-3.01 P<0.63

HR: Hazard Ratio; CI: Confidence Interval

*** not included in meta-analysis, but included here to show the analysis of different non-GTPase analgesic drugs in the study of Forget (2010).**

Supplementary Figure 1:

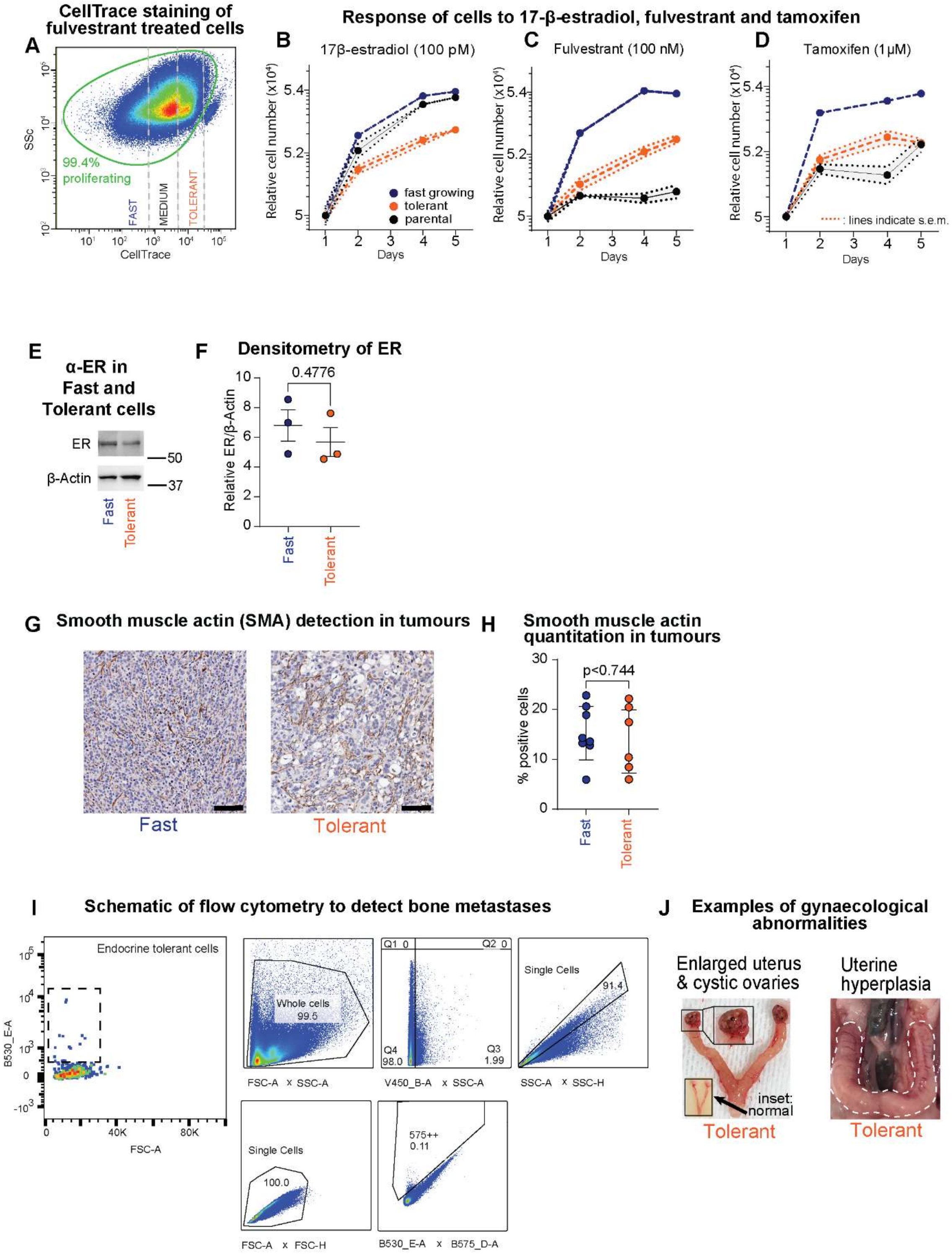

Figure S1: Supplementary Data for *in vitro* and *in vivo* studies of Fast and Endocrine Tolerant cells

(A) Two parameter flow cytometry plot of CellTrace pulsed cells (CellTrace versus side scatter (SSc)). 99.4% of cells had lost CellTrace staining (marked in orange). Dashed lines indicate partitions for sorting into low, medium and high fractions, showing the gating for Figure 1D. (B-D) Cell number of parental, Fast and Endocrine Tolerant cells following treatment with (B) 17β-estradiol (100pM), (C) fulvestrant (100nM) and (D) tamoxifen (1µM). Cell number averaged from 12 technical replicates, and data normalised between wells at time zero. Representative of 2 biological replicates. Error bars are SEM or smaller than the symbol. (E) Western blots for estrogen receptor in Fast and Endocrine Tolerant cells. β-actin used as loading control. Note that the western blots performed in these experiments were performed on the same triplicate samples as in Figure 3I, and two different exposures of the same β-Actin blot are presented in the Source Data for Figure 3I and Supplementary Figure S1E. (F) Densitometry of ER expression in Fast and Endocrine Tolerant cells normalised to β-actin from triplicate biological experiments. Error bars are SEM, analysed by two-sided unpaired t-test of n=3 replicates. (G) Representative tumours from Fast growing (n=8 Fast growing tumours) and Endocrine Tolerant (n=6 Endocrine Tolerant tumours) stained with α-SMA by immunohistochemistry. Scale bar represents 100µm. (H) Quantitation of percentage SMA positive cells within the tumour area. Data (n=8 Fast growing tumours, n=6 Endocrine Tolerant tumours) analysed by two-sided unpaired t-test, and error bars indicate SEM. (I) Flow cytometry gating strategy to identify MCF-7 cells in bone extracts, showing the gating for Figure 1N. Cells were gated for live cells using DAPI, followed by single cell discrimination, and then detection of CD298-PE. (J) Examples of gynaecological abnormalities in xenografted mice. Examples shown from animals xenografted with Endocrine Tolerant cells and include enlarged uterus and cystic ovaries, and uterine hyperplasia (dashed line) (representative of 8 instances of abnormalities). Inset is normal uterus and ovaries. Source data are provided as a Source Data file.

Supplementary Figure 2:

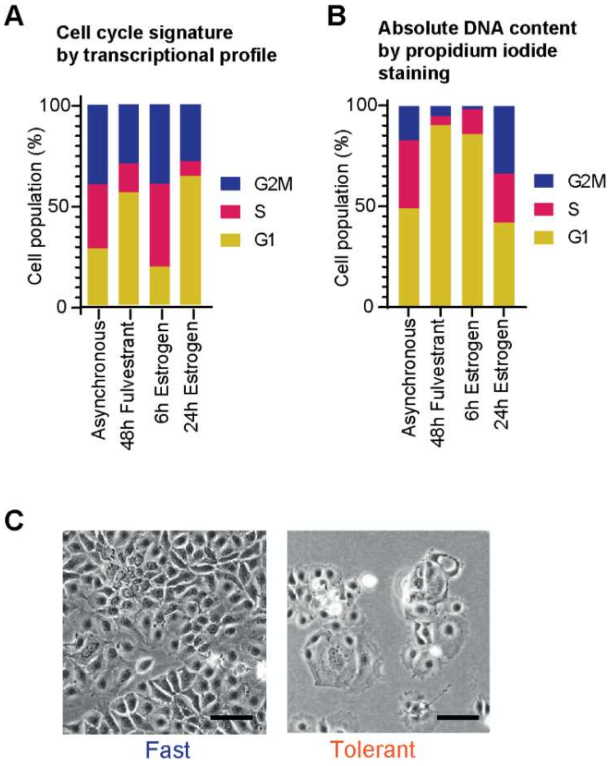

Figure S2: Supplementary Data for scRNAseq analysis

(A) scRNAseq analysed cells (asynchronous: 2336; 24h estrogen treatment: 941; 6h estrogen treatment: 2066; 24h fulvestrant treatment: 340) were separated into cell cycle fractions by transcriptional profile. G_1 phase = mustard, S phase = pink, G_2/M phase = blue. (B) Short-term fulvestrant and estrogen treated MCF-7 cells were analysed for cell cycle phase by flow cytometry using propidium iodide staining for DNA content and quantified for cell cycle distribution, >20,000 events/analysis. Representative of results previously established in [14]. G_1 phase = mustard, S phase = pink, G_2/M phase = blue. (C) Fast growing and Endocrine Tolerant cells in culture, images representative of n=3 biological replicates. Scale bar =100μm.

Supplementary Figure 3:

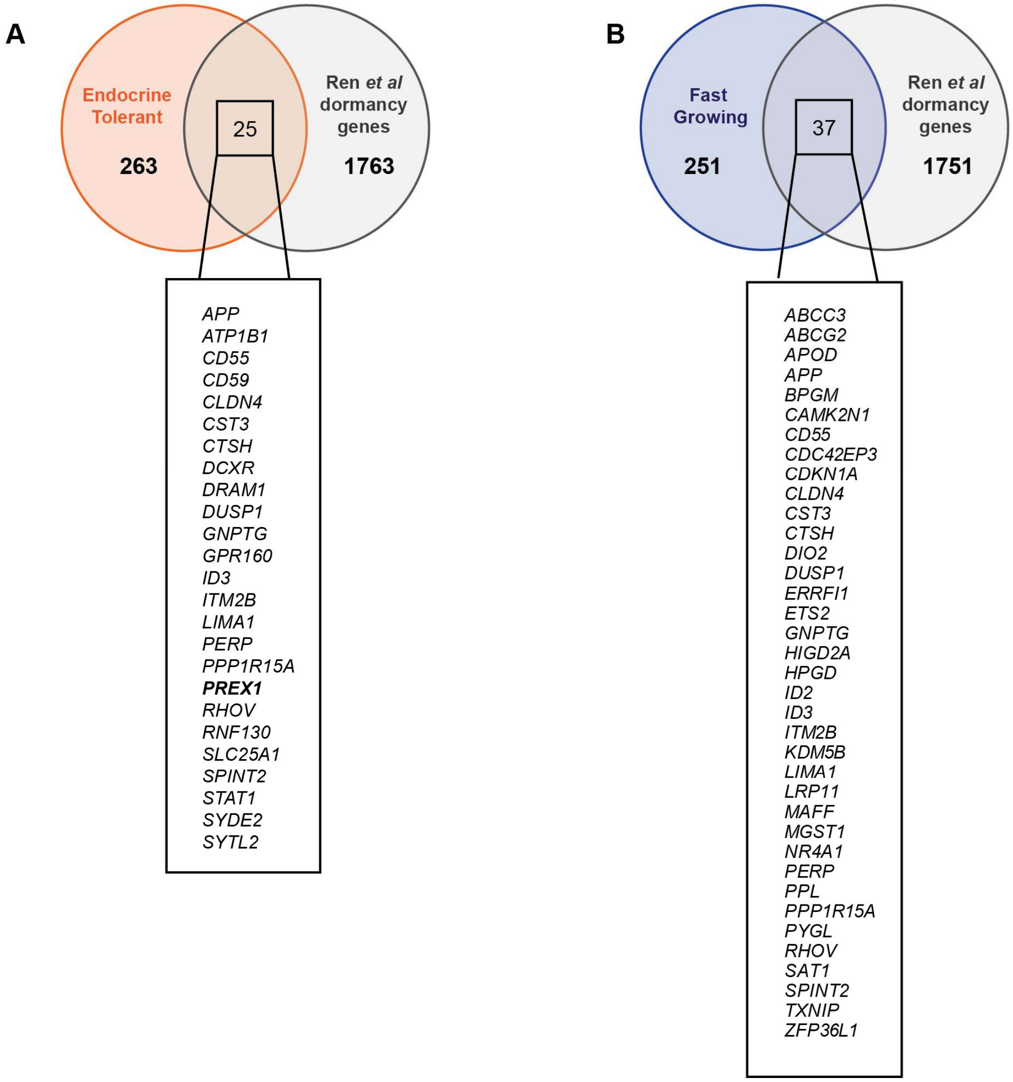

Figure S3: Comparison of gene signatures of resistance models to a dormancy gene set

(A) Venn diagram of comparison of enriched transcriptome of Endocrine Tolerant cells (n=288 genes) to genes enriched in a dormancy model of ER+ breast cancer (n=1788 genes) (26). Boxed region is overlapping genes. (B) Venn diagram of comparison of enriched transcriptome of Fast-growing cells (n=288 genes) to genes enriched in a dormancy model of ER+ breast cancer (n=1788 genes). Boxed region is overlapping genes.

Supplementary Figure 4:

Similar ERK activation in Fast Growing and Endocrine Tolerant cell lines

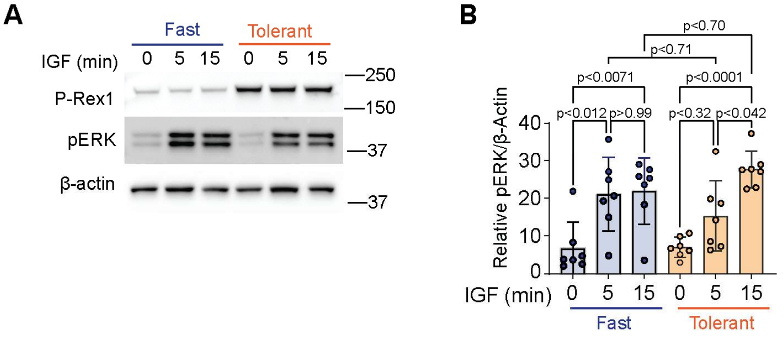

C Correlation between *EGFR* and *PREX1* mRNA in the Metabric dataset

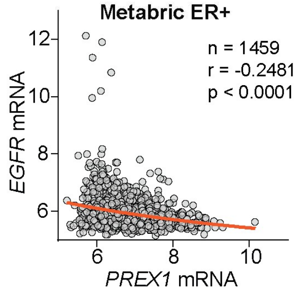

Figure S4: Supplementary data on differential signalling pathway induction

(A) Representative western blot of P-Rex1, pERK and β-actin in Fast and Endocrine Tolerant cells treated with 10ng/mL IGF1 for 5 and 15mins. (B) Densitometric analysis of Western Blots for pERK expression from (A). Data presented as mean ± SEM from seven biological replicates. Analysed by ordinary one-way ANOVA with Tukey's multiple comparison test. (C) Relationship between *PREX1* and *EGFR* expression in ER+ breast cancers of the Metabric cohort (n=1459 samples). r = Spearman's correlation analysis. Orange line is least squares fit. Source data are provided as a Source Data file.

Supplementary Figure 5:

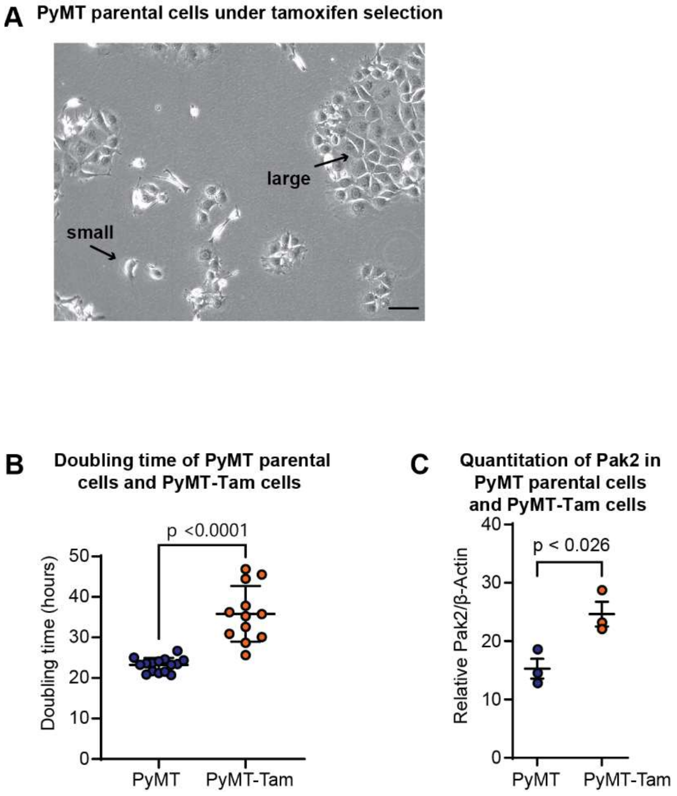

Figure S5: Supplementary Data for MMTV-PyMT cell line studies

(A) MMTV-PyMT Rac1-FRET cells under chronic exposure to 1 µM tamoxifen for 15 days. Arrows indicate small and large colonies that are visible at this early timepoint. Image representative of >3 images. Scale bar is 100µm. (B) Doubling times of MMTV-PyMT Rac1-FRET (PyMT) (n=15) and chronically tamoxifen treated MMTV-PyMT Rac1-FRET (PyMT-Tam) cells after >30 passages of drug exposure (n=12). Each replicate represents a different biological passage. Analysed by two-sided unpaired t-test, error bars are SEM. (C) Densitometry of Pak2 expression in PyMT and PyMT-Tam cells normalised to β-actin from triplicate biological experiments. Analysed by two-sided unpaired t-test, error bars are SEM. Source data are provided as a Source Data file.

Supplementary Figure 6:

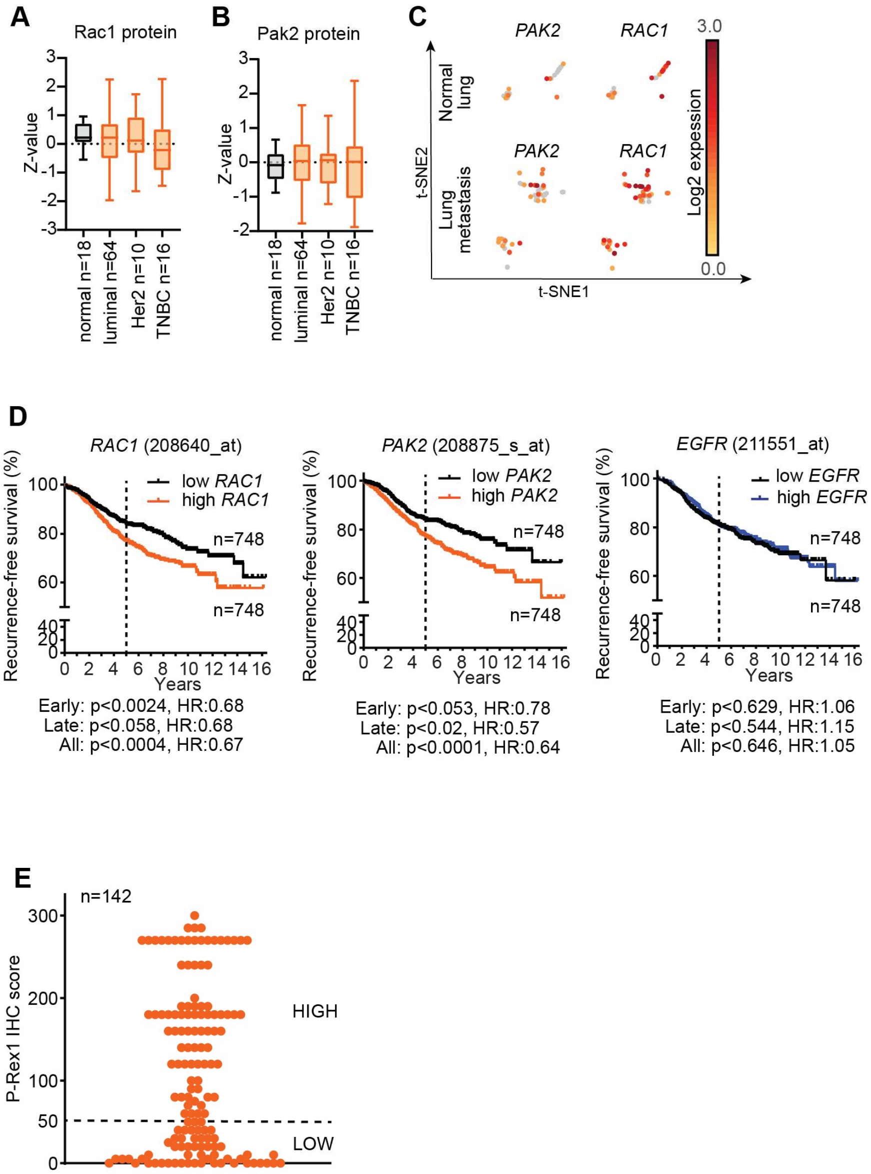

Figure S6: Supplementary Data for Figure 5

(A-B) Protein expression from CPTAC (122) across normal breast (n=18), luminal type breast cancers (n=64), Her2 (n=10), triple negative (TNBC; n =16) for (A) Rac1 and (B) Pak2. Data and statistical outcomes sourced from UALCAN (122), and t-tests analysis described in (122). (C) scRNAseq analysis of a lung metastasis (n=51 cells) and normal lung tissue (n=27 cells) from a breast cancer patient previously treated with tamoxifen, ovarian suppression, aromatase inhibitors and chemotherapies. tSNE plots shown for *RAC1* and *PAK2* within epithelial cell subsets. (D) Recurrence-free survival of patients with high (n=748) and low (n=748) *RAC1* expression (208640_at), high (n=748) and low (n=748) *PAK2* expression (208875_s_at), or high (n=748) and low (n=748) *EGFR* expression (211551_at) treated with tamoxifen and aromatase inhibitors, divided into early (0-5 yrs) and late (5-16.5 yrs) recurrence. Data analysed by Log-rank (Mantel-Cox) test with hazard ratio. (E) Distribution of H-scores of P-Rex1 IHC staining on 142 primary and metastatic samples from 54 breast cancer patients with gynaecological metastases. Dashed line indicates demarcation between high (H-score>50) and low (H-score≤50) staining samples. Source data are provided as a Source Data file.

Supplementary Figure 7:

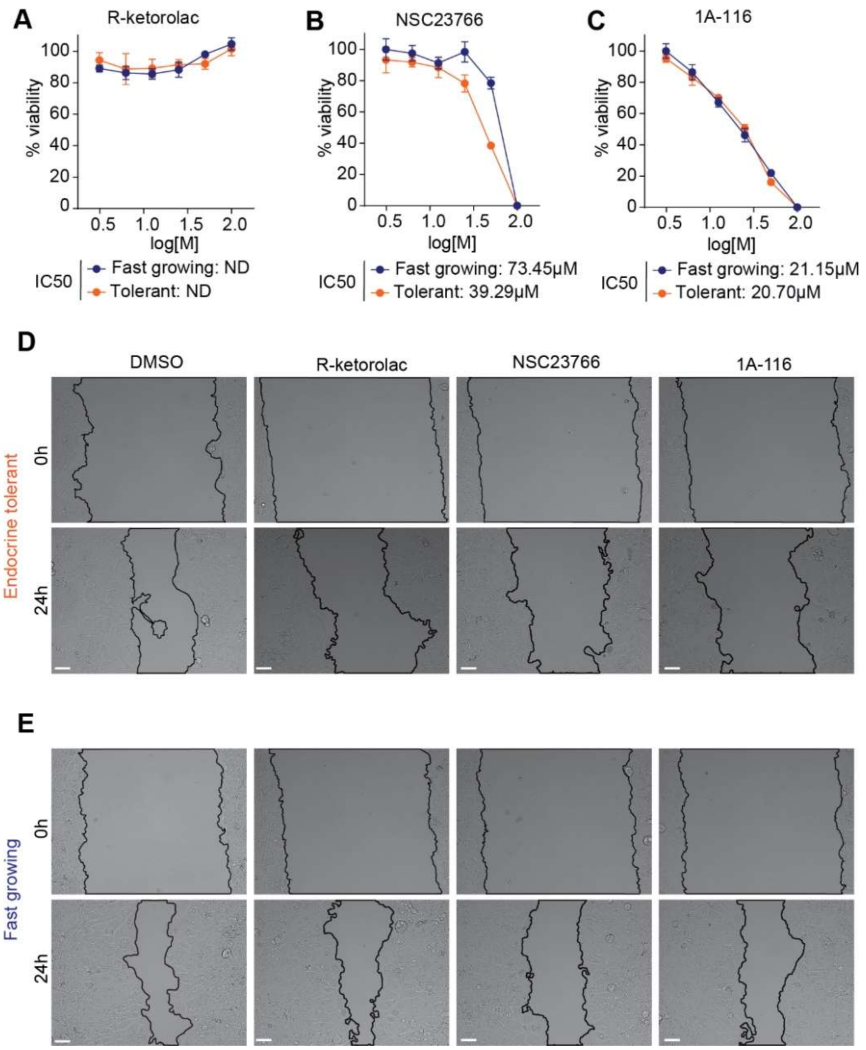

Figure S7: Supplementary Data for Figure 6

(A-C) Dose response curves and IC50 determination for (A) R-ketorolac, (B) NSC23766 and (C) 1A-116 in fast growing and endocrine tolerant cell lines from n=4 biological replicates. IC50 values could not be determined for R-ketorolac. ND = not determined. (D-E) Representative images (from n=3 biological replicates) of scratch wound assays from (D) endocrine tolerant and (E) fast growing resistant cells at 0h and 24h, treated with DMSO, 100 µM R-ketorolac, 25 µM NSC23766 and 6.25 µM 1A-116 for 24 hours. Wounds are outlined in black. Scale bar = 50µm. Source data are provided as a Source Data file.

Supplementary Figure 8:

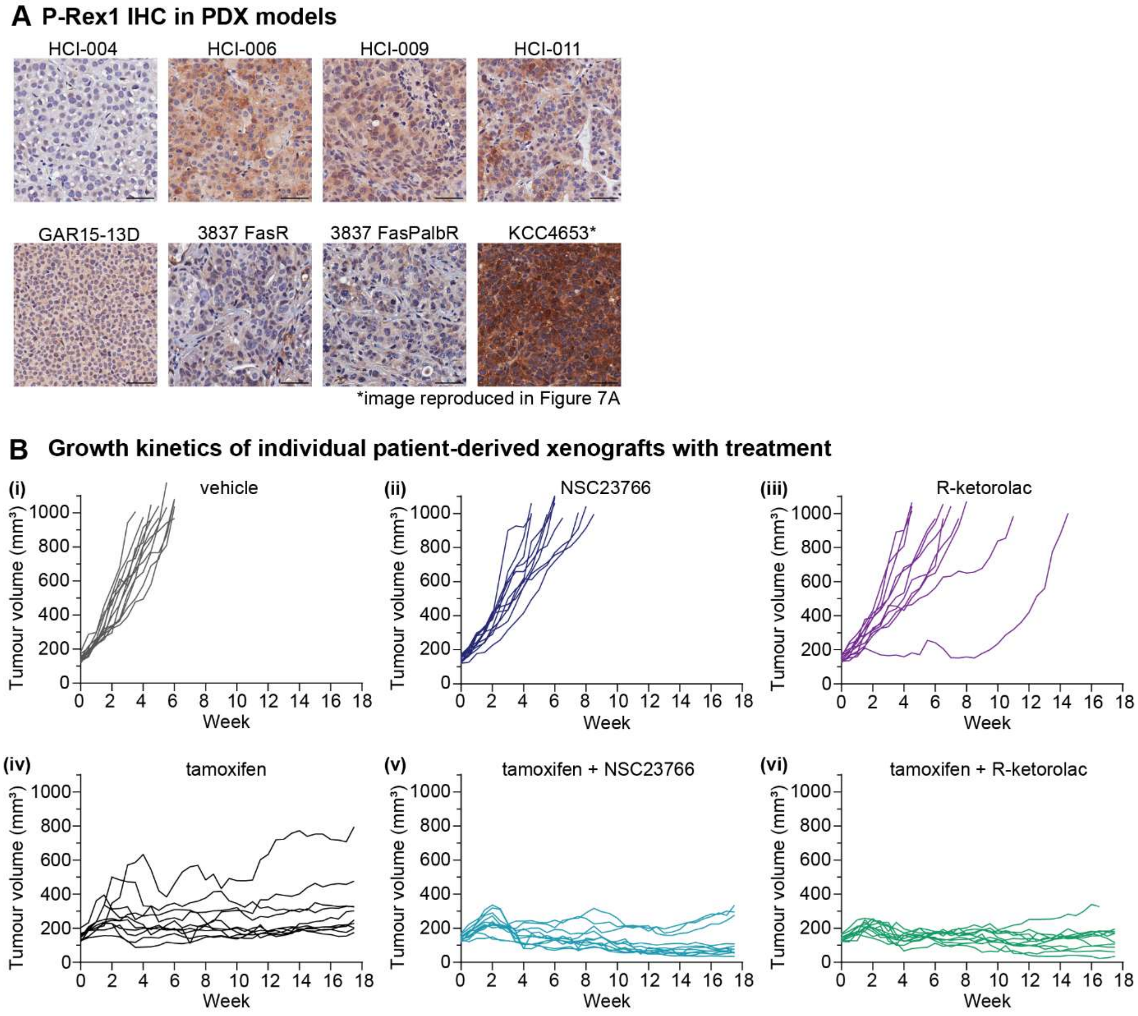

Figure S8: P-Rex1 IHC of PDX models, and growth kinetics of treatment of the KCC4653 PDX model

(A) IHC screening for P-Rex1 expression in eight tumours from patient-derived xenograft models listed in Supplementary Table 1. Scale bar = 50µm. Note that the IHC staining panel of KCC4653 shown in Figure 7A is reproduced here adjacent to other IHC staining to allow a side-by-side comparison. (B) Growth kinetics of individual KCC4653 PDX tumours exposed to different treatments. Following tumour implantation and expansion to 150mm^3, mice were treated with (i) Vehicle (n=12, 45% saline/45% PEG300/ 5% Tween 80/ 5% DMSO administered by oral gavage 5 days/week), (ii) 4 mg/kg NSC 23766 (n=11, in H_2O, intraperitoneal injection, 5 days/week), (iii) 10 mg/kg R-ketorolac (n=11, in 45% saline/45% PEG300/ 5% Tween 80/ 5% DMSO, administered by oral gavage 5 days a week), (iv) Tamoxifen (n=10, 60 day 5mg pellet), (v) Tamoxifen + 4 mg/kg NSC23766 (n=11) and (vi) Tamoxifen + 10 mg/kg R-ketorolac (n=11). Tamoxifen pellets were reimplanted halfway through treatment. Tumour growth was measured by callipers. Source data for tumours are provided in the source data for Figure 7C of the main manuscript.

Supplementary Figure 9:

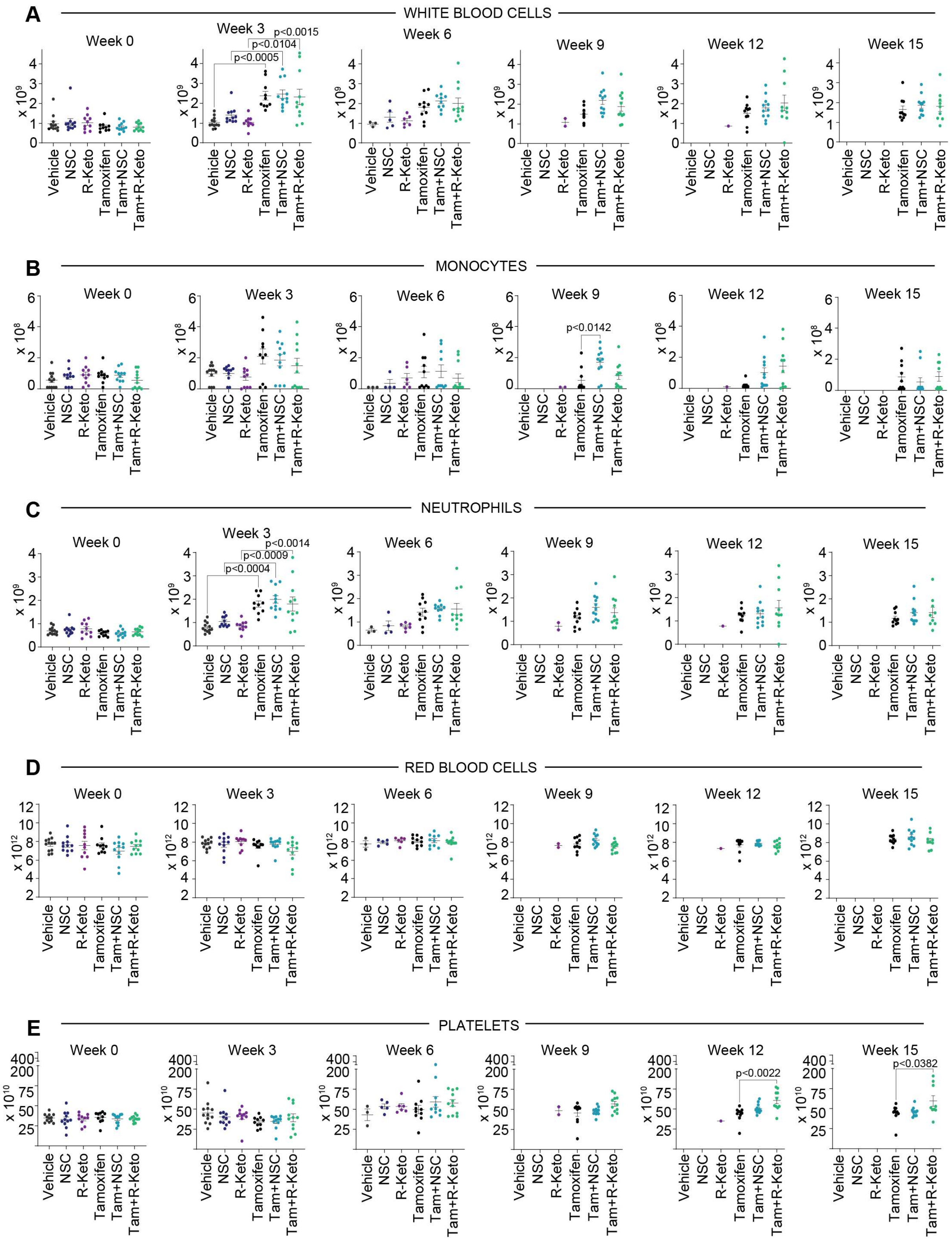

Figure S9: Toxicity studies for treatments of the KCC4653 PDX model

(A-E) Changes in peripheral blood count in response to treatments at weeks 0, 3, 6, 9, 12 and 15 of treatment with each dot representing one mouse. Changes in (A) white blood cell count, (B) monocyte count, (C) neutrophil count, (D) red blood cell count and (E) platelet count. The entire cohort of animals were analysed at each timepoint, excluding animals that had reached ethical endpoint, or samples that had technically failed in the Abaxis VetScan HM5 hematology analyser. Each sample represents one mouse, and the number of samples per measurement per timepoint is shown in the Source Data. Data was analysed using one-way ANOVA with Tukey's multiple comparisons. Source data are provided as a Source Data file.

Supplementary Figure 10:

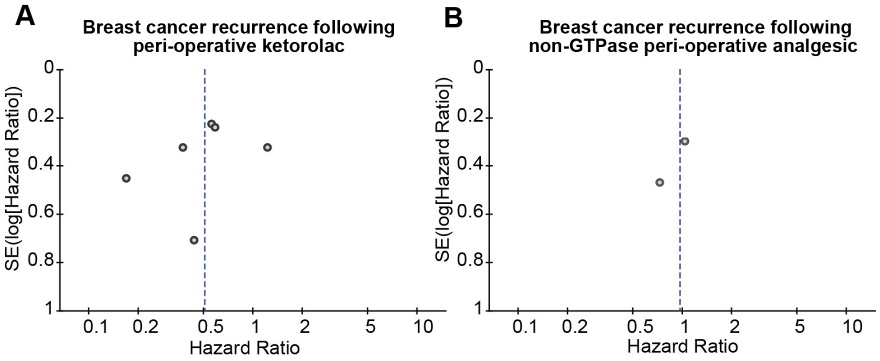

Figure S10: Funnel plots for meta-analysis

(A) Funnel plot of studies on peri-operative ketorolac (n=6) that were examined by meta-analysis. SE is standard error. Dotted line indicates overall hazard ratio from the meta-analysis. (B) Funnel plot of studies on peri-operative non-GTPase analgesics (n=2) that were examined by meta-analysis. SE is standard error. Dotted line indicates overall hazard ratio from the meta-analysis.
