# Supplementary material for: Endocrine therapy reprogramming of breast cancer facilitates metastatic escape via upregulation of P-Rex1/Rac1 signalling
Source: Nat Commun. 2026 May 11;17:3042. doi: 10.1038/s41467-026-70683-x (PMC13161276; doi:10.1038/s41467-026-70683-x)
Supplement: Supplementary file 2 — Reporting Summary [file 41467_2026_70683_MOESM2_ESM.pdf]

nature portfolio

Corresponding author(s): Caldon CE

Last updated by author(s): Nov 13, 2025

Reporting Summary

Nature Portfolio wishes to improve the reproducibility of the work that we publish. This form provides structure for consistency and transparency in reporting. For further information on Nature Portfolio policies, see our Editorial Policies and the Editorial Policy Checklist.

Statistics

For all statistical analyses, confirm that the following items are present in the figure legend, table legend, main text, or Methods section.

n/a	Confirmed	
☐	☒	The exact sample size (*n*) for each experimental group/condition, given as a discrete number and unit of measurement
☐	☒	A statement on whether measurements were taken from distinct samples or whether the same sample was measured repeatedly
☐	☒	The statistical test(s) used AND whether they are one- or two-sided<br>*Only common tests should be described solely by name; describe more complex techniques in the Methods section.*
☐	☒	A description of all covariates tested
☐	☒	A description of any assumptions or corrections, such as tests of normality and adjustment for multiple comparisons
☐	☒	A full description of the statistical parameters including central tendency (e.g. means) or other basic estimates (e.g. regression coefficient) AND variation (e.g. standard deviation) or associated estimates of uncertainty (e.g. confidence intervals)
☐	☒	For null hypothesis testing, the test statistic (e.g. *F*, *t*, *r*) with confidence intervals, effect sizes, degrees of freedom and *P* value noted<br>*Give P values as exact values whenever suitable.*
☒	☐	For Bayesian analysis, information on the choice of priors and Markov chain Monte Carlo settings
☒	☐	For hierarchical and complex designs, identification of the appropriate level for tests and full reporting of outcomes
☐	☒	Estimates of effect sizes (e.g. Cohen's *d*, Pearson's *r*), indicating how they were calculated

Our web collection on statistics for biologists contains articles on many of the points above.

Software and code

Policy information about availability of computer code

Data collection

FACSDiva (BD Biosciences) was used for flow cytometry data collection.
Multiplexed protein data was generated with Bio-Plex Manager MP (BioRad).

Data analysis

• Flow cytometry data was analyzed using FlowJo (BD Biosciences).
• Statistical analyses were performed with Excel (Microsoft) and Prism (v 10.0.0, GraphPad).
• Tumor sections were quantitated with QuPath (https://qupath.github.io/).
• Multiplexed protein data was analysed with the Bio-Plex Manager 6.1 software (BioRad).
• scRNAseq data (Figure 2) was analyzed with Seurat pipelines as described in Valdés-Mora et al Cell Reports. 2021;35(2):108945 and at https://github.com/GaldesLab/CellReportsManuscript.
• An additional scRNAseq dataset (Figure 5) was analysed with Cell Ranger software (v 3.1.0), normalised with Seurat (v.3.1.2) in R (v.3.6.1), and visualised with Loupe Browser 5.1.0.
• Publicly available scRNAseq datasets were analyzed using the Broad single cell data portal (https://singlecell.broadinstitute.org/single_cell).
• Publicly available protein expression data was analyzed via UALCAN (https://ualcan.path.uab.edu/cgi-bin/ualcan-res.pl).
• Survival data based on normalized mRNA expression was accessed via KMPlotter (https://www.kmplot.com/) and analyzed with Prism (v 10.0.0, GraphPad).
• GSEA was performed with ShinyGo (https://bioinformatics.sdstate.edu/go77). Genelists were further analysed for gene regulatory networks by using “signalling network analysis”, the Signor 2.0 database, and Steiner Forest network analysis via NetworkAnalyst (https://www.networkanalyst.ca).
• Densitometry of Western blots, second harmonic generation signal quantification, fibrillar collagen PR-Cy3 signal quantification, ß-galactosidase assay scoring, scratch wound assay quantification, colony area quantification and transwell migration quantification were carried out using ImageJ (v1.53)/FIJI (v2.16.0). Quantitative area measurement of fibrillar collagen was carried out with ImageJ/FIJI script

available via GitHub (https://github.com/TCox-Lab).
• Single cell analysis of Rac activity was performed using FLIMfit (v5.1.1)
• Hormone response assays were analysed using the IncuCyte ZOOM Software Version 2016B.
• Mus musculus gene identifiers were converted into human identifiers using g:Profiler (https://biit.cs.ut.ee/gprofiler/gost).
• Meta-analysis was performed using Revman 5.0 software. Forest plots were generated with Revman 5.0.

For manuscripts utilizing custom algorithms or software that are central to the research but not yet described in published literature, software must be made available to editors and reviewers. We strongly encourage code deposition in a community repository (e.g. GitHub). See the Nature Portfolio guidelines for submitting code & software for further information.

Data

Policy information about availability of data

All manuscripts must include a data availability statement. This statement should provide the following information, where applicable:

- Accession codes, unique identifiers, or web links for publicly available datasets
- A description of any restrictions on data availability
- For clinical datasets or third party data, please ensure that the statement adheres to our policy

Single cell datasets relating to Figure 2 have been deposited in GEO Datasets at GSE306192 [https://www.ncbi.nlm.nih.gov/geo/query/acc.cgi?acc=GSE306192]. The Figure 5 scRNAseq data generated in this study have been deposited in the EGA database under study accession EGAS00001008353 [https://ega-archive.org/studies/EGAS00001008353]. EGAS00001008353 data are available under restricted access for reasons of patient confidentiality, access can be obtained through application to the Data Access Committee. The datasets GSE43566 [https://www.ncbi.nlm.nih.gov/geo/query/acc.cgi?acc=GSE43566] and GSE176078 [https://www.ncbi.nlm.nih.gov/geo/query/acc.cgi?acc=GSE176078] which were reanalysed for this study are available at GEO datasets. The data portals KM plotter [https://kmplot.com/analysis], UALCAN [https://ualcan.path.uab.edu/analysis-prot.html] and cBioPortal [https://www.cbioportal.org] were used to access other listed publicly available datasets. Otherwise, the data supporting the findings of this study are available within the paper, or in its Supplementary Information. Source data are provided with this paper.

Research involving human participants, their data, or biological material

Policy information about studies with human participants or human data. See also policy information about sex, gender (identity/presentation), and sexual orientation and race, ethnicity and racism.

Reporting on sex and gender

The current study is on breast cancer, which occurs predominantly in women. For this reason only biological samples from women were analyzed as part of this project.
NEW STUDY:
MonARC study (described below).
RETROSPECTIVE STUDIES:
The following are retrospectively reanalyzed cohorts for which population characteristics, recruitment, and ethics oversight have already been described in the listed publications.
- Kutasovic JR, McCart Reed AE, Males R, Sim S, Saunus JM, Dalley A, et al. Breast cancer metastasis to gynaecological organs: a clinico-pathological and molecular profiling study. J Pathol Clin Res. 2019;5(1):25-39.
- Cummings MC, Simpson PT, Reid LE, Jayanthan J, Skerman J, Song S, et al. Metastatic progression of breast cancer: insights from 50 years of autopsies. The Journal of Pathology. 2014;232(1):23-31.
- Esha Jain, Jorge Gómez Tejeda Zañudo, Mary McGillicuddy, Daniel L. Abravanel, Beena S. Thomas, Dewey Kim, Sara Balch, John Navarro, Jakob H. Weiss, Tania G Hernandez, Michael Dunphy, Brett N. Tomson, Jorge Buendia-Buendia, Oyin Alao, Alyssa L. Damon, Simona Di Lascio, Shahrayz Shah, Ilan K. Small, Delia Sosa, Lauren Sterlin, Imani Boykin, Rachel E. Stoddard, Netsanet Tsegai, Ulcha F. Ulysse, Kolbe Phelps, Elizabeth Frank, Priti Kumari, Simone Maiwald, Katie Larkin, Sam Pollock, Andrew Zimmer, Parker S. Chastain, Taylor Cusher, Colleen Nguyen, Sarah Winnicki, Elana Anastasio, Eliezer M. Van Allen, Eric S. Lander, Todd R. Golub, Corrie A. Painter, Nikhil Wagle. The Metastatic Breast Cancer Project: leveraging patient-partnered research to expand the clinical and genomic landscape of metastatic breast cancer and accelerate discoveries. medRxiv 2023.06.07.23291117; doi: https://doi.org/10.1101/2023.06.07.23291117
- Cancer Genome Atlas Network. Comprehensive molecular portraits of human breast tumours. Nature. 2012 Oct 4;490(7418):61-70. doi: 10.1038/nature11412. Epub 2012 Sep 23. PMID: 23000897; PMCID: PMC3465532.
- Pereira B, Chin SF, Rueda OM, Vollan HK, Provenzano E, Bardwell HA, Pugh M, Jones L, Russell R, Sammut SJ, Tsui DW, Liu B, Dawson SJ, Abraham J, Northen H, Peden JF, Mukherjee A, Turashvili G, Green AR, McKinney S, Oloumi A, Shah S, Rosenfeld N, Murphy L, Bentley DR, Ellis IO, Purushotham A, Pinder SE, Børresen-Dale AL, Earl HM, Pharoah PD, Ross MT, Aparicio S, Caldas C. The somatic mutation profiles of 2,433 breast cancers refines their genomic and transcriptomic landscapes. Nat Commun. 2016 May 10;7:11479. doi: 10.1038/ncomms11479. PMID: 27161491; PMCID: PMC4866047.

Reporting on race, ethnicity, or other socially relevant groupings

These groupings were not used in the manuscript.

Population characteristics

Samples from a single female participant in the MONARC study were analysed in this study.

Recruitment

Participants in the MONARC study were breast cancer patients who donate cancer samples by autopsy upon their death. Patients were recruited by participating oncologists at St Vincent's Hospital, Sydney, Australia. Only a limited number of patients participated in the study, and this was the only available dataset for ER+ breast cancer, which is this topic of the current project. As a single exploratory set of samples there is a low risk of bias in recruitment, although all outcomes are limited to the observations within a single patient.

Ethics oversight	Human samples were collected through the MonARC Program, approved human ethics protocol SVH17/173 at St Vincent's Hospital, Sydney Australia, and analysed under the Personalized Medicine for Breast Cancer study, protocol x19-0496.

Note that full information on the approval of the study protocol must also be provided in the manuscript.

Field-specific reporting

Please select the one below that is the best fit for your research. If you are not sure, read the appropriate sections before making your selection.

☒ Life sciences ☐ Behavioural & social sciences ☐ Ecological, evolutionary & environmental sciences

For a reference copy of the document with all sections, see nature.com/documents/nr-reporting-summary-flat.pdf

Life sciences study design

All studies must disclose on these points even when the disclosure is negative.

Sample size	Biosensor studies were performed on n=4 animals per study. Four was chosen for sample size to minimize the use of an invasive procedure. Intraductal xenografts were performed on n=10 animals per arm. Statistical methods were not explicitly used to determine sample size prior to experiments. However, this sample size gives an ~80% power to detect a 20% change in tumour size with an SD of 15%.<br>+/- tamoxifen treatment of MMTV-PyMT mouse model was performed across a cohort of n=20 mice to achieve a similar power.<br>Patient-derived xenografts were performed on cohorts of 10-12 mice/arm to achieve a similar power.<br>In vitro studies were performed on 3-12 replicates, as indicated.
Data exclusions	Data were not excluded.<br>For the data presented in Figure 1F-1O the entire animal cohort was not analyzed in all post study experiments. Since there were only 6 palpable tumours in the Tolerant arm, 5-8 tumours from fast growing xenografts were randomly selected for comparison in Supp Fig 1H, Fig1J and Fig 1L. 1 animal was not included for post-analyses in Fig 1M, 1O as an autopsy could not be performed on this mouse. A subset of animals were analyzed for bone metastases (Fig 1N). This was because the protocol was technically developed during cohort progression and bone marrow was only successfully collected from a subset of the animals.
Replication	All biological assays were performed at least in triplicate, except where indicated. scRNAseq cell line studies were performed on 700-2000 cells/sample. As for all projects, this project required training on new equipment, in new techniques or with new reagents. Some of these attempts were technical failures and were not included in the final dataset.
Randomization	For animal experiments the animals were randomly allocated to treatment arms. For all other experiments where multiple plates of cells were used, different treatment groups were randomly allocated, or were allocated left to right in multi-well plates (eg Vehicle, Treatment 1, Treatment 2).
Blinding	A patient cohort was scored for staining for the immunohistochemistry for P-Rex1. The pathologist, E.M., scored these data blinded to patient identity and characteristics. Data analysis of the cohort was performed by an independent researcher, A. M-R, blinded to hypothesised outcome. Blinding was not used for experiments with a single or low number of technical replicates, and that were collected over time courses requiring immediate processing and analysis. These parameters made it logistically impractical to instigate a blinded approach.

Reporting for specific materials, systems and methods

We require information from authors about some types of materials, experimental systems and methods used in many studies. Here, indicate whether each material, system or method listed is relevant to your study. If you are not sure if a list item applies to your research, read the appropriate section before selecting a response.

Materials & experimental systems

n/a	Involved in the study	
☐	☒	Antibodies
☐	☒	Eukaryotic cell lines
☒	☐	Palaeontology and archaeology
☐	☒	Animals and other organisms
☐	☒	Clinical data
☒	☐	Dual use research of concern
☒	☐	Plants

Methods

n/a	Involved in the study	
☒	☐	ChIP-seq
☐	☒	Flow cytometry
☒	☐	MRI-based neuroimaging

Antibodies

Antibodies used	The following antibodies were used:<br>• Cyclin D1, https://datasheets.scbt.com/sc-20044.pdf, Santa Cruz Biotechnology, #sc-20044, (Monoclonal) DCS-6, 1:500 (WB)<br>• Cyclin A, https://datasheets.scbt.com/sc-239.pdf, Santa Cruz Biotechnology, #sc-239, (Monoclonal) BF683, 1:1000 (WB)<br>• Beta-actin, https://datasheets.scbt.com/sc-69879.pdf, Santa Cruz Biotechnology, #sc-69879, (Monoclonal) AC-15, Lot F1011,

1:15,000 (WB)
• GAPDH, https://datasheets.scbt.com/sc-32233.pdf, Santa Cruz Biotechnology, #sc-32233, (Monoclonal) 6C5, Lot A1110, 1:15,000 (WB)
• p21, https://www.bdbiosciences.com/content/dam/bdb/product_assets/product_pdf/singcolorpureantibody/purified/pdf_2/610234.pdf, BD Biosciences, #610234, (Monoclonal) 70/Cip1/WAF1, 1:1000 (WB)
• Total Rb, https://www.bdbiosciences.com/content/dam/bdb/product_assets/product_pdf/singcolorpureantibody/purified/pdf_0/554136.pdf, BD Biosciences, #554136, (Monoclonal) G3-245, 1:500 (WB)
• Rac1, https://www.cytoskeleton.com/pdf-storage/datasheets/arc03.pdf, Cytoskeleton Inc, #ARC03, (Monoclonal), 1:1000 (WB)
• PAK2, https://static.abclonal.com/datasheet/A4553.pdf, AB Clonal, #A4553, (Monoclonal) ARC1030, Lot 4000001030, 1:2000 (WB)
• P-Rex1, https://www.sigmaaldrich.com/AU/en/product/sigma/hpa001927?srsltid=AfmBOoqQFV88kiiVZLCqbcJcHoAzmT86oXPfuGNcKDrwD1b4TbMhZFM0, https://data.atlasantibodies.com/product-datasheets/hpa001927.pdf, Sigma-Aldrich (Atlas Antibodies), #HPA001927, (Polyclonal), Lot E114486, 1:500 (IHC), 1:1000 (WB)
• Estrogen receptor alpha (human) (HC-20), https://datasheets.scbt.com/sc-543.pdf, Santa Cruz Biotechnology, #sc-543, (Polyclonal), Lot J1206, 1:500 (WB)
• Estrogen receptor alpha (mouse), https://doc.abcam.com/datasheets/active/ab32063/en-us/estrogen-receptor-alpha-antibody-e115-chip-grade-ab32063.pdf, Abcam, #ab32063, (Monoclonal) E115, Lot GR3216811-7, 1:1000 (WB)
• EGFR, https://www.abnova.com.tw/upload/media/product/document/2007/DS_H00001956-M02.pdf, Abnova, #H00001956-M02, (Monoclonal) 4H2, 1:1000 (WB)
• pEGFR (Tyr1045), https://www.cellsignal.com/products/2237/datasheet?images=1&protocol=0&size=A4, Cell Signaling Technology, #2237, (Polyclonal), 1:1000 (WB)
• pHER2 (Tyr1221/1222), https://www.cellsignal.com/products/2243/datasheet?images=1&protocol=0&size=A4, Cell Signaling Technology, #2243, (Monoclonal) 6B12, 1:1000 (WB)
• HER2, https://www.cellsignal.com/products/2165/datasheet?images=1&protocol=0&size=A4, Cell Signaling Technology, #2165 (Monoclonal) 29D8, 1:1000 (WB)
• pERK (Thr202/Tyr204), https://www.cellsignal.com/products/9101/datasheet?images=1&protocol=0&size=A4, Cell Signaling Technology, #9101, (Polyclonal), 1:1000 (WB)
• pIR/IGF1R (Tyr1162/Tyr1163) https://www.thermofisher.com/order/genome-database/dataSheetPdf?producttype=antibody&productsubtype=antibody_primary&productId=44-804G&version=Local, Invitrogen, #44-804G, (Polyclonal), 1:1000 (WB)
• Smooth muscle actin, https://doc.abcam.com/datasheets/active/ab5694/en-us/alpha-smooth-muscle-actin-antibody-ab5694.pdf, Abcam, #ab5694, (Polyclonal), Lot 1038192-3, 1:100 (IHC)
• Cytokeratin, https://www.thermofisher.com/order/genome-database/dataSheetPdf?producttype=antibody&productsubtype=antibody_primary&productId=MA1-12594&version=Local, Invitrogen, #MA1-12594, (Monoclonal) C11, Lot TB2517338, 1:200 (IHC)
The Milliplex Map RTK Phosphoprotein Magnetic Bead panel – Cell Signaling Multiplex Assay – 9 Plex (HPRTKMAG01K) was also used.

Validation

ANTIBODY DETECTION OF SIGNALLING PROTEINS:
Results from the Milliplex Map RTK Phosphoprotein Magnetic Bead panel (HPRTKMAG01K) were cross validated within the manuscript using EGFR (H00001956-P02) and pHER2 (Y1221/1222, H00001956-P02) from Abnova; pEGFR (Y1045, Cat#2237), HER2 (29D8, Cat#2165) and ERK (T202/Y204 Cat#9101) from Cell Signalling; and pIR/IGF1R (Y1162/Y1163, #44-804G) from Invitrogen.
Validation antibodies performed as indicated on the datasheets.
• EGFR, https://www.abnova.com.tw/upload/media/product/document/2007/DS_H00001956-M02.pdf, Abnova, #H00001956-M02, (Monoclonal) 4H2, 1:1000 (WB)
• pEGFR (Tyr1045), https://www.cellsignal.com/products/2237/datasheet?images=1&protocol=0&size=A4, Cell Signaling Technology, #2237, (Polyclonal), 1:1000 (WB)
• pHER2 (Tyr1221/1222), https://www.cellsignal.com/products/2243/datasheet?images=1&protocol=0&size=A4, Cell Signaling Technology, #2243, (Monoclonal) 6B12, 1:1000 (WB)
• HER2, https://www.cellsignal.com/products/2165/datasheet?images=1&protocol=0&size=A4, Cell Signaling Technology, #2165 (Monoclonal) 29D8, 1:1000 (WB)
• pERK (Thr202/Tyr204), https://www.cellsignal.com/products/9101/datasheet?images=1&protocol=0&size=A4, Cell Signaling Technology, #9101, (Polyclonal), 1:1000 (WB)
• pIR/IGF1R (Tyr1162/Tyr1163) https://www.thermofisher.com/order/genome-database/dataSheetPdf?producttype=antibody&productsubtype=antibody_primary&productId=44-804G&version=Local, Invitrogen, #44-804G, (Polyclonal), 1:1000 (WB).
Data from bead panel and western blots are shown in Figure 3.

IMMUNOHISTOCHEMISTRY
The P-Rex1 antibody used in this manuscript has been characterised as ENSG00000124126 in Protein Atlas. Immunohistochemistry with the P-Rex1 antibody was validated using normal and cancer tissues as matching Protein Atlas data. Kidney tissue showed low staining as per Protein Atlas https://www.proteinatlas.org/ENSG00000124126-PREX1/tissue#expression_summary, whereas high expression is seen in ER+ breast cancers. Other IHC antibodies performed as expected from the datasheets:
• Smooth muscle actin, https://doc.abcam.com/datasheets/active/ab5694/en-us/alpha-smooth-muscle-actin-antibody-ab5694.pdf, Abcam, #ab5694, (Polyclonal), Lot 1038192-3, 1:100 (IHC)
• Cytokeratin, https://www.thermofisher.com/order/genome-database/dataSheetPdf?producttype=antibody&productsubtype=antibody_primary&productId=MA1-12594&version=Local, Invitrogen, #MA1-12594, (Monoclonal) C11, Lot TB2517338, 1:200 (IHC)

OTHER WESTERN BLOTS
For estrogen receptor detection, human specific (Santa Cruz, HC20, https://www.scbt.com/p/eralpha-antibody-hc-20) and mouse specific (Abcam, ab32063; https://www.abcam.com/products/primary-antibodies/estrogen-receptor-alpha-antibody-e115-chip-grade-ab32063.html) antibodies were used. MCF-7 cell lysate was used a positive control to identify the correct molecular weight of ER, see Source Data, Figure 4C.
P-Rex1 antibody epitope is used to detect human protein, but is also a high homology match for mouse detection (https://www.atlasantibodies.com/products/antibodies/primary-antibodies/triple-a-polyclonals/prex1-antibody-hpa001927/), and has been previously used to detect mouse P-Rex1 (https://www.biorxiv.org/content/10.1101/2022.10.24.513541v2).
Other antibodies performed as expected from the datasheets:

• Cyclin D1, https://datasheets.scbt.com/sc-20044.pdf, Santa Cruz Biotechnology, #sc-20044, (Monoclonal) DCS-6, 1:500 (WB)Cyclin A, https://datasheets.scbt.com/sc-239.pdf, Santa Cruz Biotechnology, #sc-239, (Monoclonal) BF683, 1:1000 (WB)
• Beta-actin, https://datasheets.scbt.com/sc-69879.pdf, Santa Cruz Biotechnology, #sc-69879, (Monoclonal) AC-15, Lot F1011, 1:15,000 (WB)
• GAPDH, https://datasheets.scbt.com/sc-32233.pdf, Santa Cruz Biotechnology, #sc-32233, (Monoclonal) 6C5, Lot A1110, 1:15,000 (WB)
• p21, https://www.bdbiosciences.com/content/dam/bdb/product_assets/product_pdf/singcolorpureantibody/purified/pdf_2/610234.pdf, BD Biosciences, #610234, (Monoclonal) 70/Cip1/WAF1, 1:1000 (WB)
• Total Rb, https://www.bdbiosciences.com/content/dam/bdb/product_assets/product_pdf/singcolorpureantibody/purified/pdf_0/554136.pdf, BD Biosciences, #554136, (Monoclonal) G3-245, 1:500 (WB)
• Rac1, https://www.cytoskeleton.com/pdf-storage/datasheets/arc03.pdf, Cytoskeleton Inc, #ARC03, (Monoclonal), 1:1000 (WB) Detects human and mouse protein.
• PAK2, https://static.abclonal.com/datasheet/A4553.pdf, AB Clonal, #A4553, (Monoclonal) ARC1030, Lot 4000001030, 1:2000 (WB) Detects human and mouse protein.

Eukaryotic cell lines

Policy information about cell lines and Sex and Gender in Research

Cell line source(s)	MCF-7 cells - Michigan Cancer Foundation.<br>MMTV-PyMT cell lines - derived in-house from MMTV-PyMT tumors.
Authentication	Authentication was performed using STR profiling at GMG, Garvan Institute, Australia in May 2023, and compared to human cell lines (MCF-7 cells) and mouse cell lines (MMTV-PyMT cell lines).
Mycoplasma contamination	Cell lines are routinely tested for mycoplasma contamination.
Commonly misidentified lines<br>(See ICLAC register)	None.

Animals and other research organisms

Policy information about studies involving animals; ARRIVE guidelines recommended for reporting animal research, and Sex and Gender in Research

Laboratory animals	8-12 week old immunocompromised NOD-SCID-IL2R-/- mice were used for the xenograft study. Following xenografting, animals were maintained for up to 9 months or ethical endpoint.<br>MMTV-PyMT/Rac1 FRET transgenic mice (Floerchinger A et al Cell Reports. 2021;36(11):109689) were used for the mammary imaging window studies. Studies were performed to monitor primary tumour development (0-16 weeks) or examine Rac activity in tumours (12-16 weeks).<br>8-12 week old immunocompromised NOD-SCID-IL2R-/- mice were used for the patient-derived xenograft study. Following xenografting, animals were maintained until ethical endpoint or study end (~9-10 months).
Wild animals	None.
Reporting on sex	Female mice were used exclusively for this study as it involves breast cancer, a predominantly female malignancy.
Field-collected samples	None.
Ethics oversight	Animal procedures: Garvan/St Vincent's Animal Ethics Committee (Animal ethics numbers 16/13; 17/23; 19/13; 24/16; 21/09).

Note that full information on the approval of the study protocol must also be provided in the manuscript.

Clinical data

Policy information about clinical studies
All manuscripts should comply with the ICMJE guidelines for publication of clinical research and a completed CONSORT checklist must be included with all submissions.

Clinical trial registration	No clinical trial was analysed.
Study protocol	Human samples (Fig 5E-5F) were collected through the MonARC Program, approved human ethics protocol SVH17/173 at St Vincent's Hospital, Sydney Australia, and analysed under the Personalized Medicine for Breast Cancer study, protocol x19-0496.
Data collection	Samples and data were collected in 2017-2019 at St Vincent's Hospital, Sydney Australia.
Outcomes	The study was observational without pre-defined outcomes.

Flow Cytometry

Plots

Confirm that:

☒ The axis labels state the marker and fluorochrome used (e.g. CD4-FITC).

☒ The axis scales are clearly visible. Include numbers along axes only for bottom left plot of group (a 'group' is an analysis of identical markers).

☒ All plots are contour plots with outliers or pseudocolor plots.

☒ A numerical value for number of cells or percentage (with statistics) is provided.

Methodology

Sample preparation	CellTrace staining: MCF-7 cells treated with 100nM fulvestrant for 20 days had their media removed, and were washed once with prewarmed PBS to remove all traces of serum. 2uM CellTrace was added and cells incubated in a CO2 incubator for 20 min. The CellTrace solution was removed, and cells washed 2x with medium containing at least 1% csFCS (serum binds excess CellTrace), followed by addition of culture media plus 100nM fulvestrant. Cells were cultured for a further 12 days until cell sorting. Cells were then trypsinised, and resuspended in sorting buffer (PBS +2% FCS +1.5mM EDTA + 2 x Penicillin/ Streptamycin + 2 x Antibody/Antimycotic).<br>Bone metastases: A subset of animals were analyzed for cancer cells in the bone marrow. Bone marrow from the femur and tibia of mice were flushed with PBS, additional cells collected from crushed bone, and passed through a mesh filter. Cells pellets were collected by centrifugation at 1200 rpm, for 5 mins at 4C and resuspended in 200 µL of PBS, prior to filtration with a 40 µM filter. Cells were blocked with 1 µL of FC block (BD Biosciences) for 10 mins on ice followed by incubation with PE human CD298 (BioLegend) for 20 mins. Cells were resuspended in 500 µL of FACS buffer (2% FBS in PBS), and co-stained with DAPI (0.5 µg/mL, Invitrogen).
Instrument	Cells were sorted on a FACSAria (BD Biosciences). Cells were analysed on FACS Canto II (BD Biosciences).
Software	Data was analysed with FlowJo software.
Cell population abundance	CellTrace: CellTrace staining occurs across all cells in the population, and is gated into low (10-20%) medium (40-50%) and high (10-20%) expression.<br>Bone marrow metastases: Bone marrow metastases represent a rare population of <0.1% of cells in xenografted mice. These human cells are detected in mouse bone marrow by using a human specific cell surface marker (CD298).
Gating strategy	Gating strategies for CellTrace staining is shown in Figure 1D/Supplementary Figure 1A, and bone marrow analysis in Supplementary Figure 1F.<br>CellTrace Gating: CellTrace staining of cells was visualized on FSc-A vs V450-A. Cells were sorted into Cell Trace (V450-A) low, medium and high.<br>Detection of human cells stained with PE human CD298 in mouse bone marrow: Whole cells were identified using FSc-A/SSc-A followed by live/dead selection with DAPI on V450-A vs SSc-A. FSc-A/FSc-H. Single cells were identified on SSc-A vs SSc-H and FSc-A/FSc-H. Human cells were detected by B530E-A vs B575_D-A, followed by B530_E-A vs FSc-A.

☒ Tick this box to confirm that a figure exemplifying the gating strategy is provided in the Supplementary Information.
